# Use of an e‑portfolio mapping tool: connecting experiences, analysis and action by learners

**DOI:** 10.1007/s40037-019-0514-5

**Published:** 2019-05-16

**Authors:** Sylvia Heeneman, Erik Driessen, Steven J. Durning, Dario Torre

**Affiliations:** 1 Department of Pathology, Faculty of Health, Medicine and Life Sciences, Maastricht University, Maastricht, The Netherlands; 2 Department of Educational Development and Research, Faculty of Health, Medicine and Life Sciences, Maastricht University, Maastricht, The Netherlands; 3 Uniformed Services University of the Health Sciences, Bethesda, MD USA

**Keywords:** Reflection, Concept mapping, Portfolio

## Abstract

Reflective practice is an important characteristic of a knowledgeable health professional. Reflection is needed to maintain professional competence, practice-based learning, and an improvement focused attitude. Yet, learners struggle with reflective practice activities as they are perceived as challenging and time consuming. We used the idea of concept mapping to design an e‑portfolio tool that supports learners with their reflective practice activities. The purpose of this article is to demonstrate the design and rationale of e‑portfolio mapping and how this tool could be used to support reflective practice activities.

Following the stepwise approach typical for the reflective cycle, two types of maps were designed, a trigger map and a competency map. In a trigger map, the learner reflects on a concrete learning or feedback experience. In a competency map the learner interprets and synthesizes several previous trigger maps which ultimately leads to the formulation of new learning objectives. The e‑portfolio mapping tool can be a useful and supportive tool to foster learners’ reflective skills and provide mentors with in-depth insight into the students’ learning and reflection processes.

**Electronic supplementary material:**

The online version of this article (10.1007/s40037-019-0514-5) contains supplementary material, which is available to authorized users.

## Introduction

Reflective practice is an essential skill for health professionals. The ability to reflect on one’s experiences is critical to identify learning needs, maintain professional competence, and enhance practice-based learning and improve attitude over time [1, 2]. Reports on the use of reflection for learning and professional development have been mixed. Reflection has been shown to foster self-regulated learning, i.e. a deliberate, cyclical process whereby self-generated thoughts, feelings, and actions are planned and adapted to attain learning goals [3]. On the other hand, reflection is also perceived by learners as a meaningless ritual and time-consuming process, which does not fulfil its purpose [4]. There is a clear need for a system (e.g. an educational tool) that aligns both with the theory and models of reflection and at the same time supports learners in their reflective activities in daily practice.

Several well-known reflection models are structured as cycles that include: a series of questions on what happened, the identification of feelings and thoughts, an analysis of what occurred, and steps leading to action [5, 6]. However, the use of more conventional reflection learning tools such as storytelling, critical incident analysis, or a reflective essay [7] often does not fit the sequential cyclic structure of these reflection models.

Therefore, we sought to enhance and innovate the scaffolding of reflection through integrating the cyclic, stepwise approach of the models of Gibbs and Nguyen et al. [5, 6] with the framework of concept mapping. Concept maps are a graphic representation of a set of interrelated concepts that allow learners to demonstrate knowledge integration, thereby promoting reflection and making meaning of their experiences [8]. Concept maps have been shown to improve learners’ understanding of concepts, link new ideas to existing cognitive structures, and foster critical thinking and reflection [9]. Thus, we designed a mapping tool, based on concept mapping ideas, which was incorporated into an electronic (e)-portfolio. This mapping tool follows the cycle of reflection, thereby aiming to support the nature and structure of the reflective process.

## Methods

### Design and approach

In collaboration with educationalists and a computer technician, using an existing e‑portfolio platform, we developed a reflective tool, based on concept mapping ideas, which would allow students to create maps containing text elements, connected by arrows with the option of linking words. It was intended as a form of graphic organizer to integrate assessment and feedback information in order to create meaning, facilitate understanding, and foster reflective learning [8]. Therefore, the mapping tool was designed to follow the stepwise approach typical of the reflective models [5, 6]. The model includes the following steps:

- The learner looks back on a critical incident, or a trigger such as received feedback, assessment results, or a personal experience,
- The learner analyses essential aspects of the experience,
- The learner decides on an alternative to approach the incident or acts upon the feedback, and
- The learner then re-enters the cycle.

**Fig. 1:**
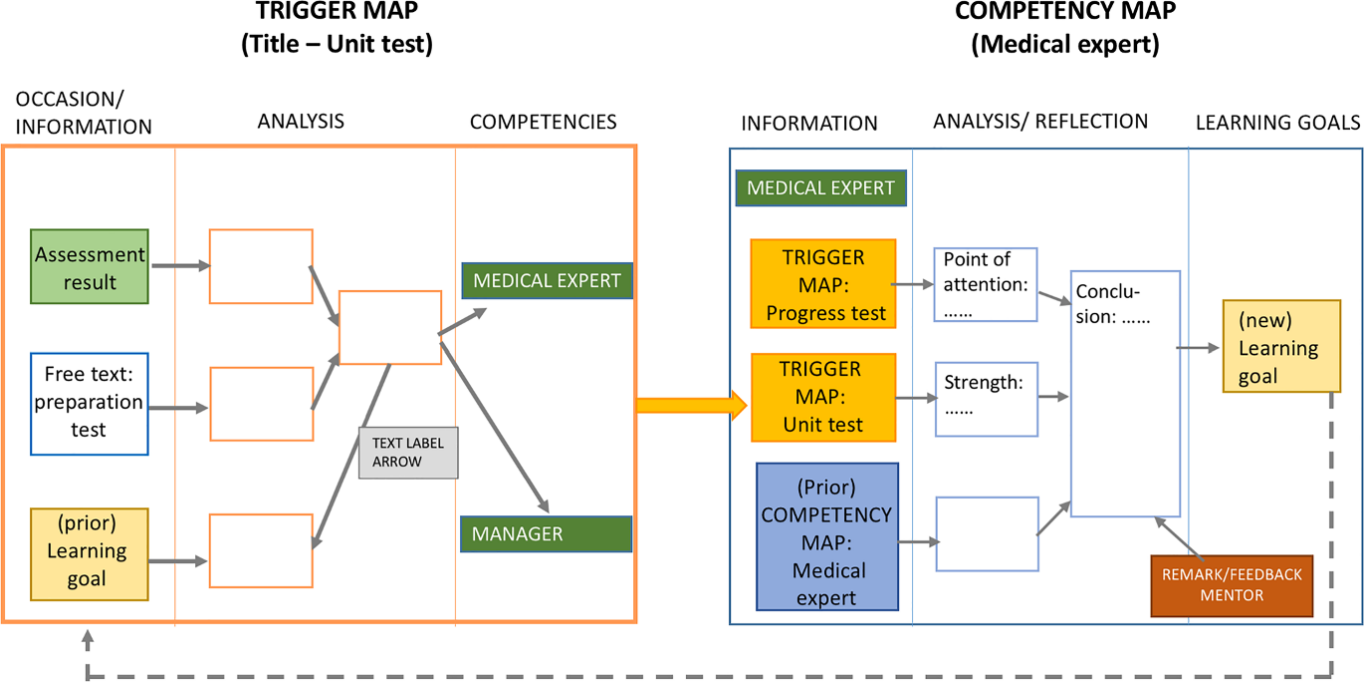
Schematic of the structure of a trigger (left) and competency map (right panel)

The tool was designed for a medical course that has a competency-based teaching and assessment program using the CanMEDS framework [10]. Therefore, in the design of the tool, it was accommodated that learners could reflect on their personal triggers, feedback etc., and relate these reflective activities to the competencies. This was done by the development of two types of ‘maps’, a trigger map and a competency map. Figure 1 shows a simple representation of the maps and how the trigger and competency map relate to each other.

The purpose of the *trigger map* is to identify the experience, i.e. the ‘trigger’ that starts the reflective cycle. The trigger map is structured in three sections (Fig. 1, left panel) and these sections guide the learner through the reflection cycle. Triggers can be a variety of experiences such as feedback that was received, an assessment result, a critical incident, or any other personal experience. The learner selects a trigger (in the occasion/information section), reflects on this trigger using the mapping tool instruments (in the analysis section in the middle) and links the map to one or more competencies (in the competency section).

The purpose of the *competency map* is to (i) foster competency development through reflection, and (ii) generate specific learning goals (Fig. 1, right panel). The structure of the competency map is comparable to the trigger map (three sections) with an important exception. The information section of the competency map contains trigger maps and prior competency maps, selected by the learner, as entry points for further reflection. This allows learners to make connections among maps and to ultimately create an interconnected network for critical analysis and reflection and, if needed, construction of the learning goal. As shown in the example of a competency map of a Year 1 learner in the figure in the online Supplementary Electronic Material, these maps are characterized by a summary of findings, and determining strength and points for improvement, which will enable the learner to detect similarities or patterns in a subsequent cycle of reflection, and action plan.

### Use of the e‑portfolio mapping tool in mentoring processes and evaluation

The e‑portfolio mapping tool was introduced in 2016 in the graduate entry course Physician-Clinical Investigator, of Maastricht University, the Netherlands, which is a 4-year medical course (*n* = 50 students/year). Learners collected a multitude of assessment and feedback information in their e‑portfolios and used this for reflection and learning in the maps. The portfolio-based reflective activities were embedded in a mentoring program where each learner had a personal mentor for 4 years [11]. The learner met with a mentor (face-to-face meetings) at regular intervals. Prior to the meeting, the learner created a number of maps, typically 6–8 trigger maps, and depending on the time interval, a number of competency maps. There were requirements for the competency maps (e.g. that each competency was the topic of reflection at least twice a year).

The mentor included comments and provided feedback prior to the meeting. All changes were saved, thus both the learner and mentor kept track of the iterative process. The maps were then discussed in the upcoming mentor meeting.

The first three meetings (of five meetings in total) in Year 1 were tailored to familiarizing the learners with analysis and reflection on their learning experiences, along with the use of the mapping tool (in a ‘learning-by-doing’ style, directed by the mentor) [11]. We intentionally did not make requirements for a specific layout for the construction of the maps. Our goal was to provide learners with the opportunity to use the mapping tool to scaffold their reflections in a way that was meaningful and relevant to them.

The e‑portfolio itself is evaluated routinely as part of program evaluation efforts at the end of the year. The evaluation is based on the objectives of the portfolio and mentoring, i.e. reflection and learning, the collection of feedback, and guidance by the mentor. For the purpose of this paper, only the information relevant for the use of the mapping tool is presented. In the Netherlands, the use of routinely collected program evaluation data for publication purposes is allowed and exempt from ethical approval.

## Results

For the purpose of this paper, we present a first impression based on the feedback of learners (Year 1, 2016–2017, *n* = 50, 56% response) and mentors (*n* = 17 total, response 58%).

Students and mentors were asked about the value of reflection using a five-point Likert scale. Students responded to the statement: ‘The mapping tool helped me in the reflective process’: with a 3.0 ± 1.1 (mean ± standard deviation).

In the narrative comments, students stated that:

- ‘The use of the maps and the ability to add information is very helpful for the reflection’;
- ‘Useful that everything is there in the portfolio and can be used and dragged in the maps for the reflection’;
- ‘The mapping system is nice. Useful that you can label the arrows’.

Mentors reported a mean score of 3.8 ± 0.8 on the helpfulness of the mapping tool. Mentors also indicated that the maps gave structure, facilitated the process of feedback, and were helpful in following the order of events and the underlying thoughts and considerations of students.

Conversely, students stated that the mapping exercise was time-consuming and some students thought that the relative importance of the reflective activities in the program should be reconsidered. Students mentioned technology difficulties, which hampered the user-friendliness of the program. Finally, some students stated they felt more comfortable with writing an essay than constructing a map.

## Discussion

We used the idea of concept mapping to design an e‑portfolio mapping tool that follows the cyclic models of reflection to support learners in developing their reflective practice activities. Although more data needs to be collected, the first evaluation suggested that the mapping tool can be helpful in reflective activities. The mentors were more positive when compared with the learners. Learners’ feedback also indicated a potential overemphasis on portfolio reflective activities resulting in increased time demands. Given our positive experiences with the mapping tool, the tool was introduced in another course in our institute, the Bachelor of Medicine.

Reflective activities were perceived by the students as time-consuming; this is in line with other reports [4, 12]. The use of the mapping tool as such will not change that and can be seen as a limitation.

We recommend providing some training, but also allowing the learners to learn and experience the benefits of the mapping tool during their self-initiated practice. Reflective learning is a personalized activity and learners should benefit most if they have sufficient agency for individualized learning [13, 14]. In the mapping tool, learner agency is fostered by the ability to structure and connect maps as is seen fit by the learner with feedback and assessment results. In addition, the first step is the choice of a meaningful personal experience or feedback by the learner. Mentors have been identified as a vital prerequisite for the guidance of reflection and a positive learning outcome for the learners [15]. Mentors need to be aware of their role in helping learners to use reflective practice meaningfully [16].

This paper has several limitations. First, we describe the experiences of a single institution, although the e‑portfolio mapping tool is now used in another course at this institution. Second, an in-depth analysis (e.g. qualitative or quantitative inquiry methods) of the outcomes and perceptions of the learners and mentors should be performed.

Given our initial experiences with the mapping tool, initial recommendations for implementation in other contexts would include tailoring reflective activities to meet learning and assessment needs of the program and individual learners. This would include the time that is spent on these activities, along with planning for the full benefits of guidance and supervision by mentors. Given the competency-based foundation of the teaching and assessment program in our medical course, we distinguished two types of maps which both used the mapping tool. Other choices for the design can be made, a single type of map may be fit for purpose for other courses. An important insight and key message from the design and first implementation is that reflective activities need to be embedded in the learning activities of the learner. The mapping tool can be useful and supportive to foster and assess reflective skills, yet should follow a fit for purpose model when implementing in other courses or programs.

In conclusion, the e‑portfolio mapping tool may aid in connecting experiences, reflection, and learning. Additionally, the tool may help mentors in understanding and providing guidance to students with the learning process.

## Caption Electronic Supplementary Material

Supplemental Figure (online): This competency map is a translated (from Dutch) map of a Year 1 student, on the competency ‘Manager’. This map contains feedback of the mentor (the red rectangle at the right side of the map). The student gave permission to use and translate the map. The text is slightly adapted for readability.
