# Supplementary figures and images for: Use of an e‑portfolio mapping tool: connecting experiences, analysis and action by learners
Source: Perspect Med Educ. 2019 May 16;8(3):197–200. doi: 10.1007/s40037-019-0514-5 (PMC6565639; doi:10.1007/s40037-019-0514-5)

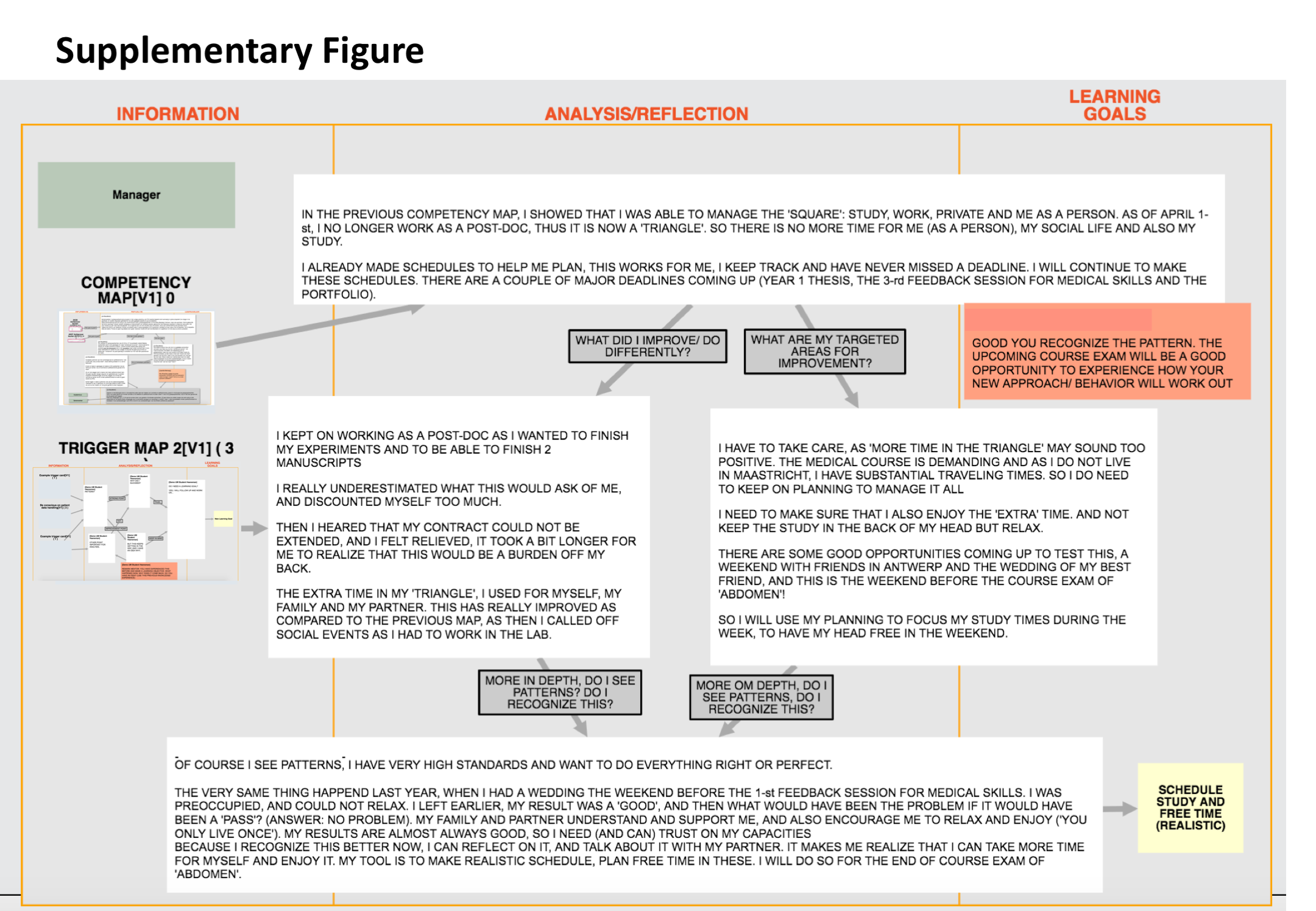

Supplement: Supplementary file 1 — Supplemental Figure (online): This competency map is a translated (from Dutch) map of a Year 1 student, on the competency ‘Manager’. This map contains feedback of the mentor (the red rectangle at the right side of the map). The student gave permission to use and translate the map. The text is slightly adapted for readability. [file 40037_2019_514_MOESM1_ESM.tiff]
